# Artificial Intelligence Meets Nail Diagnostics: Emerging Image-Based Sensing Platforms for Non-Invasive Disease Detection

**DOI:** 10.3390/bioengineering13010075

**Published:** 2026-01-08

**Authors:** Tejrao Panjabrao Marode, Vikas K. Bhangdiya, Shon Nemane, Dhiraj Tulaskar, Vaishnavi M. Sarad, K. Sankar, Sonam Chopade, Ankita Avthankar, Manish Bhaiyya, Madhusudan B. Kulkarni

**Affiliations:** 1 Department of Electronics & Telecommunication Engineering, Shri Sant Gajanan Maharaj College of Engineering, Shegaon 444203, Maharashtra, India; tpmarode@ssgmce.ac.in (T.P.M.); vkbhangdiya@ssgmce.ac.in (V.K.B.); sgnemane@ssgmce.ac.in (S.N.); dptulaskar@ssgmce.ac.in (D.T.); 2 Department of Computer Science and Engineering (IOT), Ram Meghe Institute of Technology and Research, Badnera 444701, Maharashtra, India; vmsarad@mitra.ac.in; 3 Department of Artificial Intelligence and Data Science, Vel Tech High Tech Dr. Rangarajan Dr. Sakunthala Engineering College, Avadi, Chennai 600062, Tamil Nadu, India; 4 School of Computer Science and Engineering, Ramdeobaba University, Nagpur 440013, Maharashtra, India; chopadesa@rknec.edu; 5 Symbiosis Institute of Technology, Nagpur Campus, Symbiosis International (Deemed University), Pune 411057, Maharashtra, India; ankita.avthankar@sitnagpur.siu.edu.in; 6 EQIQAI Systems Private Limited, K. V. Rangareddy, Hyderabad 500075, Telangana, India; 7 Department of Electronics and Communication Engineering, Manipal Institute of Technology, Manipal Academy of Higher Education (MAHE), Manipal 576104, Karnataka, India

**Keywords:** nail image analysis, artificial intelligence, machine learning, deep learning, non-invasive diagnosis, dermatology, smartphone-based health monitoring, explainable AI, point-of-care diagnostics

## Abstract

Artificial intelligence (AI) and machine learning (ML) are transforming medical diagnostics, but human nail, an easily accessible and rich biological substrate, is still not fully exploited in the digital health field. Nail pathologies are easily diagnosed, non-invasive disease biomarkers, including systemic diseases such as anemia, diabetes, psoriasis, melanoma, and fungal diseases. This review presents the first big synthesis of image analysis for nail lesions incorporating AI/ML for diagnostic purposes. Where dermatological reviews to date have been more wide-ranging in scope, our review will focus specifically on diagnosis and screening related to nails. The various technological modalities involved (smartphone imaging, dermoscopy, Optical Coherence Tomography) will be presented, together with the different processing techniques for images (color corrections, segmentation, cropping of regions of interest), and models that range from classical methods to deep learning, with annotated descriptions of each. There will also be additional descriptions of AI applications related to some diseases, together with analytical discussions regarding real-world impediments to clinical application, including scarcity of data, variations in skin type, annotation errors, and other laws of clinical adoption. Some emerging solutions will also be emphasized: explainable AI (XAI), federated learning, and platform diagnostics allied with smartphones. Bridging the gap between clinical dermatology, artificial intelligence and mobile health, this review consolidates our existing knowledge and charts a path through yet others to scalable, equitable, and trustworthy nail based medically diagnostic techniques. Our findings advocate for interdisciplinary innovation to bring AI-enabled nail analysis from lab prototypes to routine healthcare and global screening initiatives.

## 1. Introduction

In the quickly evolving field of digital health care, the human nail is being revealed as an unusual but uncharted window to the world of health of the entire man and of the skin. Nails also have been a cosmetic object and have a considerable amount of diagnostic information to give, inasmuch as they brace in themselves early manifestations of defects varying from starvation to autoimmune diseases and tumours [1,2,3,4]. Because nail changes are easily visible, non-invasive to test for, and photos can be taken with regular cameras or phones, a nail-based test represents a promising, low-cost way to expand digital health solutions, especially in remote or resource-poor areas [5,6,7].

AI/ML have transformed medical imaging for diagnostics within radiology, pathology, and even dermatology [8,9,10,11]. But nails remain a vastly underexplored area. Instead of depending on a doctor’s expertise and specialized equipment, AI today can identify minute shape and color changes in nail images—sometimes even those that the human eye might miss. These developments are timely, especially as the global health ecosystem shifts toward preventive care and remote patient monitoring [12,13,14,15].

Nails may provide important clues to health by reflecting signs of various medical conditions [16,17,18]. One of the most common nutritional disorders in the world, iron-deficiency anaemia, allows nails to become pale or spoon-shaped, medically known as koilonychia, reflecting a low haemoglobin state [19,20,21,22]. Onychomycosis, or fungal infection of the nail bed and plate, results in thick, discoloured, and crumbling nails and ranks among the common reasons for dermatology consults [23,24,25,26]. Psoriasis, an immune-related disease, may also involve the nails, with pitting, ridges, or separation of the nail from the nail bed, changes that are useful in early diagnosis and in monitoring the severity of the disease [27,28,29,30]. Lastly, melanoma of the nail, though rare, is an aggressive cancer that can first appear as dark streaks or pigmented spots under the nail. Early detection is critical because delayed diagnosis often leads to poor outcomes [31,32,33].

**Figure 1:**
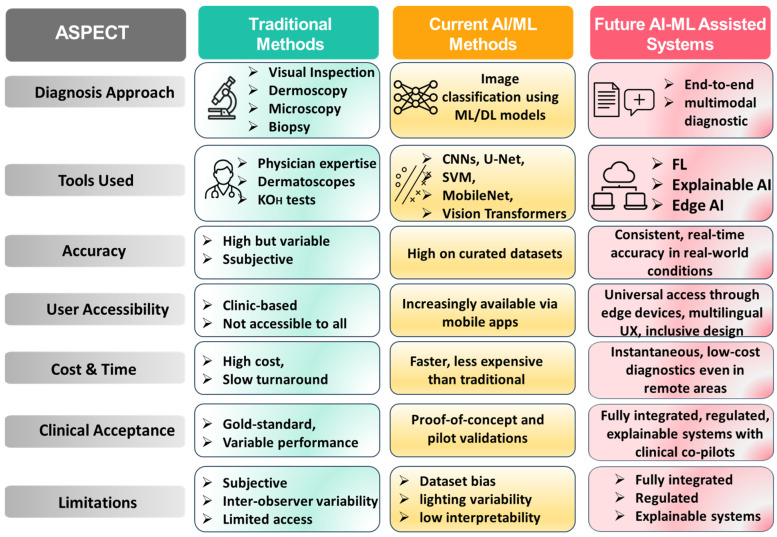
Comparative timeline of nail diagnostics: from expert-driven tools to current AI models and future integrated, explainable, and accessible AI-driven systems.

Traditional methods for diagnosing nail disorders usually rely on direct observation with or without the use of instruments, such as dermoscopy, or even laboratory tests like Potassium Hydroxide (KOH) for fungal infections and biopsy. The techniques are widely employed in clinical settings, though they have several drawbacks: they depend on the competence of a trained practitioner and might be invasive or take a lot of time to produce results. Second, variations in the results obtained from different doctors often occur. As a matter of fact, many under-resourced settings lack such facilities. Other difficulties arise with manual scoring systems, like the Nail Psoriasis Severity Index, based on subjective judgment, and changes in the disease are not always consistent. Figure 1 presents a comparison framework showing how diagnosis is shifting from traditional techniques to AI/ML-based approaches and further to more advanced AI-assisted systems [17,34,35,36,37,38].

This is exactly where AI/ML can make a real difference. They can receive nail images that detect detailed features, including changes in color, textures, and shapes, using computer vision. Certain deep learning (DL) approaches like Convolutional Neural network (CNN) have already been successful in the detection of nail disorders by recognizing particular patterns, discoloration, and lesions [39,40,41,42]. Examples include the diagnosis of iron-deficiency anemia by photographs taken with a smartphone, in which the accuracy is as high as 95%. Other models such as CNNs and U-Net, show areas affected by psoriasis on the nails. Newer and lightweight models, such as MobileNet and Vision Transformers are being developed that will allow these programs to run directly on mobile devices, creating opportunities for easy and cheap health-checks for individuals and communities [43,44,45].

This review is unique because it focuses solely on AI/ML use in nail diagnostics, a topic that is little addressed, compared to the more general studies in skin diseases and medical imaging. This review aims to bridge this gap by considering four major topics: (i) nail diseases for which AI has been used; (ii) imaging techniques and preprocessing techniques dedicated to nail data; (iii) machine learning pipelines consisting of hand-crafted and deep features; (iv) deliver options, ranging from mobile applications to real-time clinical ones. It outlines some important hurdles: (a) very few standardized nail image sets exist; (b) models should be generalizable for populations and different conditions of image acquisition; (c) it is a hard task to render them acceptable to a clinical environment. It points out the importance of precision, interpretability and the need for physicians’ trust in real world applications [2,5,17,31]. By synthesizing the expertise of fields including public health, dermatology, and computer vision, this work has set out a path for future progress in the area of non-invasive diagnosis. To sum it up briefly, nails represent a wealth of health knowledge which is underused, readily available and has excellent visual and clinical significance. The tools which are provided by AI and ML will make available the health advantages which this field offers, and this review will highlight the existing methodologies, their applications both in the present and future, the problems and opportunities which exist and call for a sharing of the knowledge which will facilitate the passing of diagnostic aids from images of nails from the laboratory into everyday use in personal monitoring health care diagnostics.

## 2. Literature Search Strategy and Study Selection

To maintain transparency, reproducibility, and a low selection bias, an a priori structured literature search strategy (based on the philosophy behind systematic review) was utilized. Though this article is a narrative technology review (vs. a PRISMA compliant systematic review), all steps for identification, screening, and selection were based on predefined rules that will guarantee adequate coverage of all published research involving AI/ML approaches to imaging-based nail diagnostics. The reviewed literature covers such aspects as imaging modalities (smartphone photography, dermoscopy, Optical Coherence Tomography (OCT), hyperspectral imaging), pre-processing pipelines, classical and deep learning models, and disease-specific applications: diabetes, hemoglobin estimation, psoriasis, anemia, melanoma, and onychomycosis.

### 2.1. Databases Searched

A multi-database strategy was used to capture both biomedical and engineering-focused research:

- PubMed/MEDLINE—clinical and dermatology-focused literature
- Scopus—multidisciplinary scientific indexing
- Web of Science Core Collection—high-quality global research coverage
- IEEE Xplore—AI/ML, computer vision, and imaging methodology papers
- Google Scholar—supplementary search to identify grey literature and emerging conference papers

Literature published from January 2000 to December 2025 was searched. This period corresponds with the emergence and rapid development of modern computer vision and DL. Reviewing of sources was done by all authors independently, who systematically screened, selected, and assessed each source based on predefined criteria to uphold objectivity and maintain academic rigor.

### 2.2. Search Keywords and Boolean Strategy

To encompass variations in terminology across dermatology, imaging, and AI, a structured Boolean search string was used: (“nail” OR “fingernail” OR “nail plate” OR “nailfold” OR “onychomycosis” OR “melanonychia”) AND (“artificial intelligence” OR “machine learning” OR “deep learning” OR “computer vision” OR “image analysis” OR “segmentation” OR “classification”) AND (“diagnosis” OR “screening” OR “prediction” OR “non-invasive detection”).

Additional disease-specific searches were conducted to capture domain-focused studies:

- “nail + diabetes + CNN/ML/AI/capillaroscopy”
- “nailbed + hemoglobin estimation + smartphone”
- “nail psoriasis + U-Net/transformer models + NAPSI scoring”
- “nail melanoma + dermoscopy + segmentation + ABCDEF rule”
- “onychomycosis + image-based diagnosis + CNN/CapsNet”

Reference lists of key papers were manually scanned to identify additional relevant studies.

### 2.3. Eligibility Criteria

**Inclusion Criteria: Studies were included if they met the following conditions:**

(A) Used AI, ML, DL, or computer vision techniques in analyzing nail images or nail-derived signals.
(B) Reported work involving nail-based diagnosis, screening, risk assessment, feature extraction, or disease monitoring.
(C) Employed validated imaging modalities, including smartphone images, dermoscopy, OCT, hyperspectral imaging, or histopathology WSIs.
(D) Published in peer-reviewed journals or reputable conference proceedings.
(E) Written in English.
(F) Provided performance metrics (accuracy, AUC, F1, RMSE, Dice score, etc.) or methodological details relevant to model interpretation.

**Exclusion Criteria:**

- Editorials, position articles, viewpoint papers without technical content
- Studies unrelated to nail imaging or diagnostics
- Papers using AI for non-image nail data only (chemical analysis without imaging)
- Veterinary or non-human nail studies
- Duplicate records
- Papers lacking sufficient methodological detail or unavailable full text

## 3. Image Acquisition Techniques for Nail Analysis

AI-assisted diagnosis of the nails is initiated with the capture of clear, high-quality images that capture all important visual details necessary for analysis. The type of adopted imaging method largely determines the accuracy, reliability, and widespread applicability of AI models. Further, we discuss the main techniques of capturing nail images, starting from regular tools such as smartphone cameras to specialized ones like dermoscopy and optical coherence tomography. All of them have certain strong and weak points in view of the AI diagnostic process [46,47].

### 3.1. Imaging Modalities

For AI to function optimally in nail diagnostics, it foremost requires good-quality images that capture the right details. Different imaging methods provide different levels of clarity, depth, and medical information, with each useful for specific clinical or technical purposes. In the ensuing sections, we discuss main ways in which nails are imaged, from simple smartphone photographs to advanced tools such as dermoscopy and OCT. Newer methods include hyperspectral and thermal imaging. For each approach, we consider its strengths, its limitations, and the extent to which it can be used effectively with AI systems.

#### 3.1.1. Smartphone Cameras

**Figure 2:**
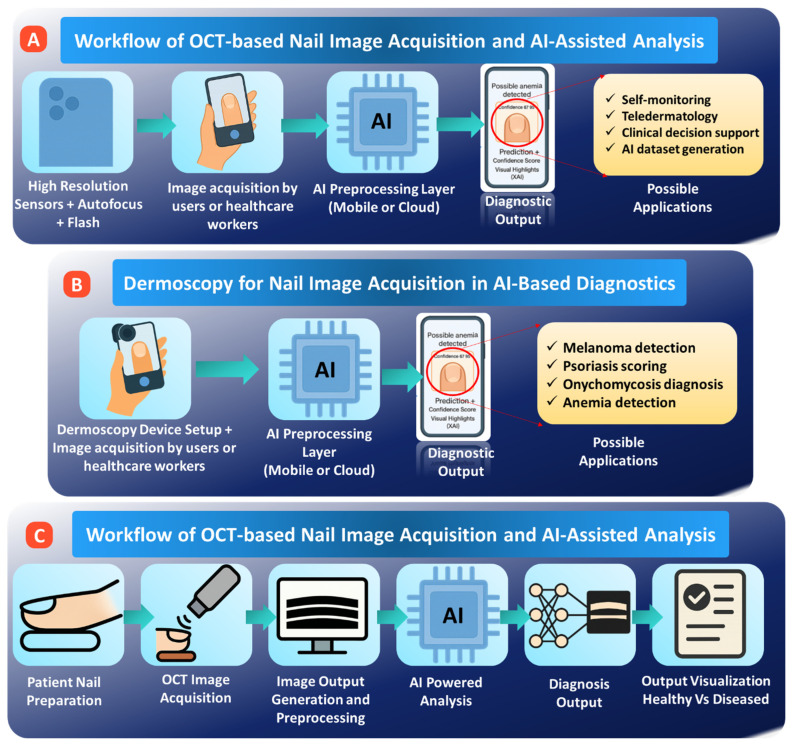
Comparative workflows of AI-assisted nail image analysis using different imaging modalities: (**A**) smartphone-based imaging, (**B**) Dermoscopy-assisted acquisition, and (**C**) OCT-based high-resolution imaging. Each pipeline highlights the steps from image acquisition and AI preprocessing to diagnostic output and potential clinical applications such as anemia detection, psoriasis scoring, and onychomycosis diagnosis.

Smartphone cameras have emerged as the most accessible modality for AI-based nail diagnostics, driving the feasibility of collecting vast amounts of data at scale and weaving imaging insights into telemedicine workflows. Modern smartphones can provide resolution that is sufficient for viewing clinically relevant features of the nail, including color variations, texture irregularities, and the boundaries of lesions shown, in Figure 2A [48,49]. However, in contrast to dermoscopy or OCT, smartphone imaging is inherently variable. Sensor design differences, color processing, image compression, lighting conditions, backgrounds, and user-driven factors related to the angle, distance, and presence of shadows-all contribute to substantial heterogeneity. This heterogeneity might dampen AI model performance if not properly addressed.

From this, researchers and clinicians are developing device-agnostic preprocessing pipelines that can normalize inputs from any type of smartphone camera; designing robust segmentation methods capable of delineating the various structures of the nails under different conditions; and creating lightweight, efficient AI models that can operate on unconstrained, real-world data. Although natural variability in these factors creates significant challenges, the advantages of smartphone-based imaging are quite apparent. The imminent feasibility of improved imaging due to the commoditization of cameras for most smartphones places this methodology in a very favorable position for translating AI diagnostics into the community environment and enabling home-based screening [50,51].

#### 3.1.2. Dermoscopy

Dermoscopy offers magnified, polarized illumination that significantly enhances the visibility of subsurface nail structures (see Figure 2B). It thus presents a particularly valuable modality for AI-based analyses, ranging from pigmentation patterns to vascular changes and early signs of melanoma [52,53]. In comparison with smartphone imaging, dermoscopy provides more standardized and diagnostically rich images due to less noise and higher clarity of features, which is important in tasks such as lesion segmentation and risk scoring. Nevertheless, dermoscopic imaging still reveals variability in optics of devices, polarization modes, operator technique, and reflectance artefacts from the nail plate itself. Given these factors, the requirement is for specialized preprocessing steps and model designs for stability across a wide range of devices and clinical settings. Despite such challenges, dermoscopy remains a clinical gold standard in evaluating nail tumors, psoriasis-related changes, and pigmented streaks, thus providing high value as an input source to train advanced diagnostic models [54,55].

#### 3.1.3. Optical Coherence Tomography (OCT)

Optical Coherence Tomography allows for high-resolution cross-sectional imaging of not only the nail plate but also the surrounding tissues, giving insight into structural features not achievable by either surface photography or dermoscopy [56,57]. These are particularly important in the diagnosis of nail diseases because the depth-resolved information obtained with OCT increases the possibility of detecting subsurface abnormalities, such as fungal invasion, psoriatic changes, and early tumor growth, which can be invisible in shallower imaging modalities, see Figure 2C. For AI applications, OCT enjoys consistent illumination and fewer surface artefacts, but it also presents several challenges. These include speckle noise inherent to the imaging process, limited penetration in nails that are thickened or swollen, and variability across different commercial OCT systems. Addressing these challenges requires specifically tailored, advanced denoising, segmentation, and feature-extraction approaches developed for volumetric data or sequential B-scan slices constituting OCT datasets. Although OCT is not as easily available in a routine clinical scenario as smartphones or dermoscopy, the ability of this modality to provide quantitative structural biomarkers makes it particularly valuable in the development of sophisticated AI models for the detection and characterization of complex or early-stage nail pathologies [58]. A detailed comparison of the technology is given in Table 1 below.

#### 3.1.4. Additional Imaging Techniques

##### Infrared (IR) Imaging

IR is a noncontact, noninvasive imaging modality that records radiation either reflected from or emitted by tissues within the near- and mid-infrared spectrum. Wavelengths utilized in IR imaging extend deeper than those of visible light, allowing sub-surface nail structures such as the nail bed, the blood vessels underneath, and even changes in chromophores to be visualized. IR imaging has therefore indicated fair potential for the assessment of changes in skin perfusion, oxygenation, and inflammatory processes relevant in disease states, including anaemia, microvascular dysfunction accompanying diabetes, and various inflammatory diseases of the nail. IR is practically advantageous in that this imaging system has less susceptibility to surface reflection and lighting variability, which allows for more stable inputs into the AI models that require consistent imaging conditions. Recent reports also underscore the potential use of near-IR for the quantification of differences in haemoglobin-related absorption occurring beneath the plate [59,60,61,62].

##### Thermal Imaging (Thermography)

Thermography is a special branch of IR imaging that works by mapping how temperature varies across the surface of the nail and the adjacent tissues. It does this by detecting the infrared radiation of long-wave length that is normally emitted by the skin surface. Since local temperature is a reflection of the underlying blood flow, metabolic activity, and inflammatory processes, thermography yields biomarkers with meaningful physiological significance. With regards to the nails, patterns of higher or lower temperature can signal microvascular problems associated with conditions such as diabetes, Raynaud’s phenomenon, or inflammatory states like psoriasis and onycholysis. Research associated with the use of AI to analyze thermal signatures for the differentiation between normal and pathological perfusion states has also been performed. It holds great promise for non-contact screening, particularly in telemedicine and home-based monitoring contexts. In contrast to RGB imaging, thermal imaging is insensitive to variation in ambient lighting and provides dynamic physiological information complementary to the structural information available with other imaging modalities [63,64,65,66,67].

##### Speckle Contrast Imaging (Laser Speckle Contrast Imaging, LSCI)

LSCI is an optical technique for measuring blood flow through the analysis of speckle patterns arising from the scattering of coherent laser light by moving red blood cells. In practice, LSCI provides real-time, high-frame-rate maps of blood perfusion over the nailfold and nail bed, enabling the quantification of microvascular reactivity, flow velocity, and capillary density. This method is widely used in research on microcirculation and is becoming increasingly relevant in the diagnostics of nails, particularly in conditions involving diabetes-associated vascular dysfunction, Raynaud’s phenomenon, systemic sclerosis, and various circulatory disorders. Combined with AI frameworks, LSCI thus allows for automatic, quantitative assessment of flow-related metrics-such as speckle contrast, perfusion indices, and flow variance-which are difficult or impossible to obtain from visual inspection alone. Because of its physiological sensitivity and excellent temporal resolution, LSCI serves as a powerful complement to structural imaging techniques [68,69,70].

##### Blood-Flow Measurement Techniques in the Nailbed

A number of optical and video-based techniques have, therefore, been developed to quantify blood flow in the nailfold and nail bed as a way of providing functional biomarkers reflecting systemic vascular health. These approaches provide an expanded view of microvascular function beyond what static images offer, helping to illuminate subtle changes in perfusion over time.

**Laser Doppler Flowmetry:** This technique represents a measurement of the Doppler frequency shift generated by erythrocytes moving within the microvasculature. Using these frequency shifts, LDF allows for the evaluation of blood flow on the basis of volume and vascular reactivity. Clinical applications have included its use in the study of diabetic microangiopathy and peripheral vascular disease through the nail area for the purpose of gaining insight into finger-level microcirculation [71,72,73].

**Video Densitometry/Capillaroscopy Flow Estimation:** Advanced nailfold capillaroscopy systems currently provide high-resolution video of capillary networks. AI algorithms can then extract quantitative features such as the velocity of capillary flow, movement patterns of red blood cells, and loop perfusion characteristics. These flow-related metrics strongly relate to conditions such as diabetic neuropathy, hypertension, autoimmune diseases, and overall cardiovascular risk and hence would be valued for functional assessment [74].

**PPG on the Nailbed: The** pulse volume changes detected through optical sensors or through smartphone-based PPG measurements at the site of the nailbed may be used as an indirect proxy for arterial stiffness, tissue perfusion, and oxygenation levels-all variables relevant to anemia and diabetes detection [75,76].

These flow-sensitive techniques complement each other in delivering functional data that extends AI-driven nail-based diagnostics beyond what is achievable from static imaging. They enable the detection of perfusion abnormalities very early and have the potential for use as digital biomarkers for longitudinal monitoring.

### 3.2. Preprocessing Strategies

After capturing images of the nails from the various devices, preprocessing is done. This preprocessing ensures that the images are cleaned up by removing noise, normalizing the images, while emphasizing the important features that contribute ultimately to diagnoses. Preprocessing ensures that the AI models are trained on a good-quality dataset that is proper and useful. This step helps in removing variability due to lighting, device type and how the user handles the camera. The following sections will cover the important preprocessing steps that enhance the accuracy, robustness, and clinical applications of the AI models implemented, such as color correction, noise reduction, normalization, extraction, and segmentation.

#### 3.2.1. Color Correction, Denoising, Normalization

Preprocessing is a very important step in AI/ML systems for nail image analysis, as images can significantly differ depending on the device, environment, and user. The most important methods include color correction, denoising, and normalization [77,78]. Of these, color correction is particularly important as many conditions of the nails are defined by their color, such as paleness of the nails in cases of anemia, yellowish colouration of the nails in fungal infections, or dark spots in melanoma. Techniques, such as modification of the white balance, histogram equalization, and algorithmic corrections can therefore help in restoring the proper color irrespective of differences in lighting or devices [79,80]. Denoising removes random artifacts caused by poor lighting, sensor noise, or compression that may mask fine details such as ridges or pits critical for diagnosis [77,81]. Commonly used filters include Gaussian or median smoothing filters, etc., and deep-learning denoisers. Normalization ensures that images from different sources will have consistent brightness and intensity important for training robust models and especially for the use of transfer learning with pre-trained networks [82,83]. These steps together enhance the accuracy, reliability, and generalizability of the AI models by making them focus on real disease features and not on irrelevant variations [84,85]. There are still challenges, including but not limited to, the curved or reflective nail surface, variations in skin tone, and background interference [86,87]. Potential future means of overcoming low-quality inputs might involve adaptively preprocessing data using AI and further enhancing it with synthetic data. In conclusion we can see that preprocessing is not simply a technical step, it is a clinical necessity for trustworthy AI-assisted nail diagnostics [88,89].

#### 3.2.2. Cropping and ROI Extraction (Nail Plate, Lunula)

ROI extraction, generally targeting the nail plate, lunula, or nail bed, is one of the most important steps in analyzing nail images after color correction and noise reduction [90,91,92]. The presence of background elements, such as skin, fingers, or shadows, contributes to noise and may decrease the accuracy of the model, hence it is essential to separate the nail region. Manual cropping is not practical for large quantities of data; therefore, automated methods will be preferred. Simple techniques involve edge detection, contour analysis, and some advanced ones with the Hough Transform algorithm, morphological operations, or color thresholding to segment the nail [93,94]. Accurately extracted ROIs mean better data quality and less overfitting, thus supporting consistent data augmentation. In the future, adaptive ROI methods based on lighting conditions, skin tone, and nail anatomy can help make models more universal, while real-time ROI extraction in smartphone apps or dermoscopic devices will simplify workflows. Overall, ROI extraction is one of those crucial steps that provide reliable, efficient, and explainable AI in nail diagnostics [95,96].

#### 3.2.3. Segmentation Techniques (U-Net, Thresholding, Edge Detection)

**Figure 3:**
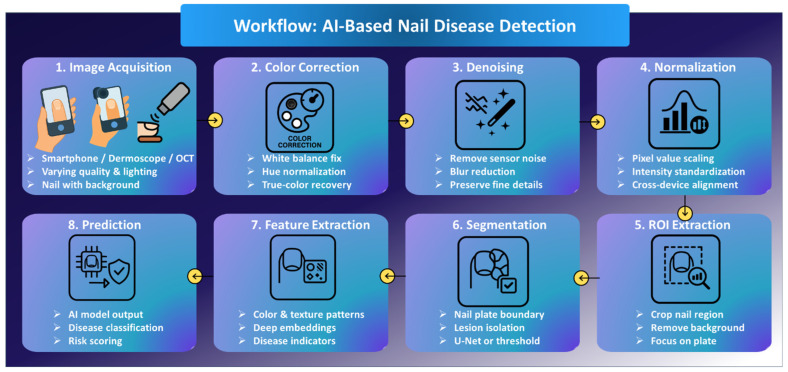
End-to-end workflow of AI-based nail disease detection.

Segmentation plays a critical role in the pre-processing step for nail image analysis, as it precisely separates the nail plate from surrounding regions such as skin, cuticles, and background [97]. Unlike Cropping or ROI extraction, segmentation offers finer detail, something quite important for conditions such as psoriasis, where surface irregularities must be localized, or melanoma, in which lesion boundaries need accurate mapping. Conventional methods such as thresholding and edge detection are simple and efficient but mostly fail under real-world conditions with irregular textures, shadows, or deformed nails [98,99]. Recently, to address such challenges, deep learning models, especially U-Net and its variants, have found widespread adoption [100]. U-Net can yield highly accurate masks that outline the nail even in the presence of poor lighting or complex cases, therefore improving the diagnostic accuracy by making sure AI models focus only on the nail region. These segmentation masks allow for the extraction of shape-based features, such as curvature or surface area, useful in the diagnosis of nail disorders. Overall, segmentation enhances both the precision and reliability of AI-assisted nail disease detection [101,102]. A stepwise visual summary of the complete AI pipeline, from image acquisition to disease prediction, is presented in Figure 3.

Lastly, preprocessing techniques such as color correction, de-noising and normalising are essential for improving image quality, lowering variability and providing consistent input for AI models when analysing the nails. ROI extraction and segmentation further isolate the diagnostically relevant nail areas ensuring that models can focus on the specific disease related information more accurately. Overall, these steps contribute to the accuracy, robustness and clinical importance of the diagnostic AI systems. A comparative overview of the techniques is presented in Table 2.

## 4. Most Used AI/ML Models for Nail-Based Diagnostics

After we have quality, preprocessed images, the next significant factor in successfully applying AI-assisted nail diagnostics is the relevant AI ML model identification and use. The AI ML models extract visual pattern features, interpret them, and classify disease conditions based on observed representations. Depending on data availability and task complexity, both classical ML algorithms and deep learning architectures have been deployed. We begin this section by discussing classical AI/ML models that have shown promise in early-stage nail analysis and diagnostic prototyping.

### 4.1. Classical ML (Feature-Based Models)

**Figure 4:**
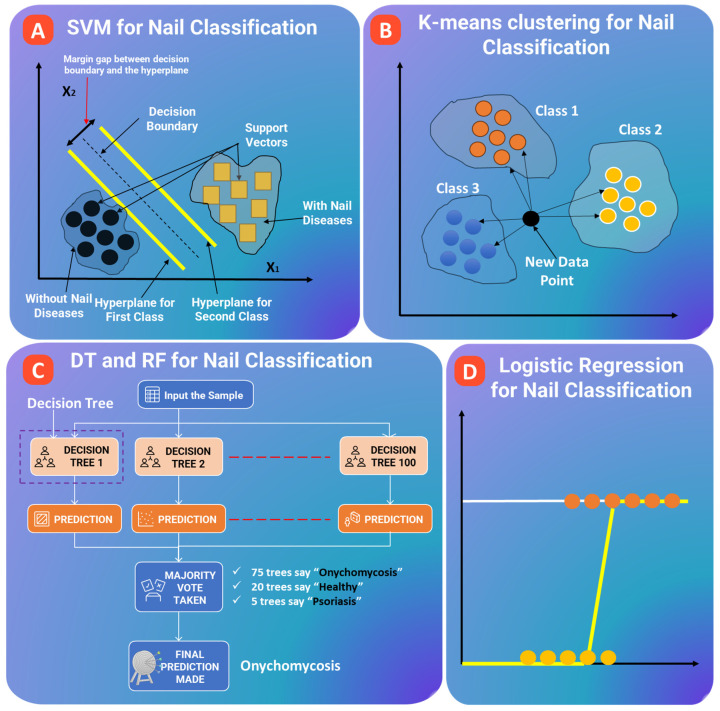
Comparative visualization of four machine learning algorithms used for nail disease classification. (**A**) Support Vector Machine (SVM) identifies the optimal hyperplane separating healthy and diseased nail samples based on support vectors. (**B**) K-means Clustering groups data into clusters (Class 1, 2, and 3), assigning new samples based on proximity to centroids. (**C**) Decision Tree and Random Forest (DT & RF) ensemble models classify input by aggregating predictions from multiple decision trees via majority voting. (**D**) Logistic Regression estimates the probability of disease presence using a sigmoid curve applied to nail features.

Most of the researchers working with small nail-image datasets still rely mostly on classical ML models, instead of newer deep learning approaches. Methods such as Support Vector Machines (SVM) (see Figure 4A) [103,104,105,106,107], k-Nearest Neighbors (kNN) (see Figure 4B) [108,109], Random Forest (RF), as shown in Figure 4C [110,111], and Logistic Regression (see Figure 4D), remain common choices. In the domain of nail diagnostics, these traditional models typically operate on simple, handcrafted features. Examples of such features are color changes indicative of anemia or hemoglobin levels, texture patterns related to conditions such as psoriatic pitting or fungal roughness, and shape/edge details indicative of melanonychia stripes or onycholysis. Statistical patterns captured from nailfold capillaroscopy, like loop density or tortuosity, also serve as informative cues for the analysis. Because those features are easy to define and interpret, classical ML models showed good accuracy in early nail-related research, especially on tasks like anemia detection or estimating the severity of psoriasis [112,113,114].

### 4.2. Deep Learning Models for Nail Analysis

**Figure 5:**
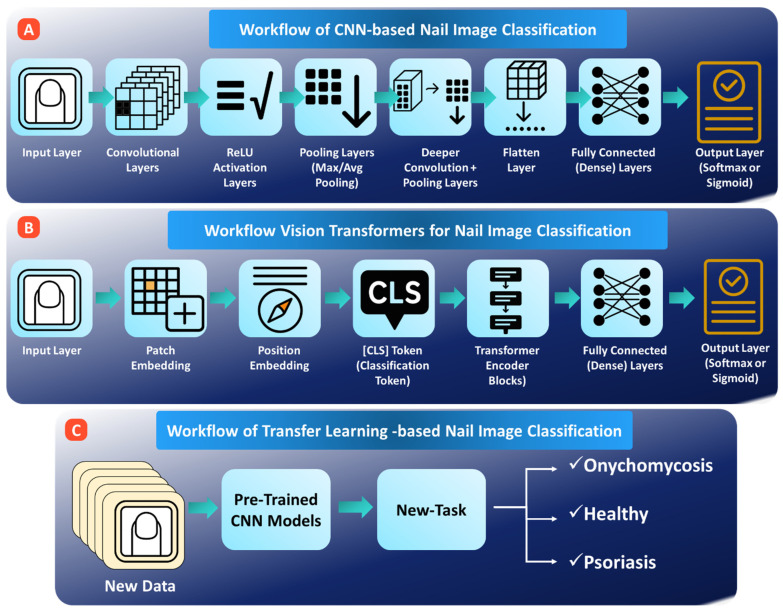
Comparative workflows of three deep learning approaches for nail image classification. (**A**) The CNN-based pipeline includes convolution, ReLU activation, pooling, flattening, fully connected layers, and a final softmax/sigmoid output layer for disease prediction. (**B**) The Vision Transformer (ViT) pipeline processes input images via patch embedding, positional encoding, and transformer encoder blocks, using a [CLS] token for classification. (**C**) The transfer learning workflow utilizes pre-trained CNN models adapted to new tasks, enabling efficient multi-class classification of nail conditions like onychomycosis, healthy nails, and psoriasis.

Deep learning methods, especially CNN [115], take center stage in diagnosing nail images by automatically learning these subtle, disease-relevant features that hand-crafted descriptors may miss [116,117,118]. CNN-based models (see Figure 5A) can detect microtexture changes in psoriatic nails, identify morphological irregularities in melanonychia lesions, pick up fine chromatic gradients linked to hemoglobin levels or diabetes-associated vascular status, and interpret the complex nailfold patterns seen in capillaroscopy. Practical smartphone-based screening tools are enabled by lightweight architectures like MobileNet, with deeper ones such as DenseNet and EfficientNet being used in more advanced clinical imaging environments to further improve the quality of results. Throughout fine-grained visual recognition tasks, these deep learning models have consistently outperformed traditional machine learning approaches [119,120,121].

### 4.3. Vision Transformers (ViTs)

The applications of vision transformers (see Figure 5B) within nail diagnostics are progressively showing very strong potential. This is especially so in tasks related to psoriasis scoring, the detection of multi-nail conditions, and those that decidedly benefit from the ability to reason on global spatial relationships throughout the whole hand. The attention mechanism in these models gives them the capability to take into consideration the full-hand context-an aspect that greatly enhances their detection capabilities regarding abnormalities diffused across multiple nails and also a reduction in errors that occur due to analysis in cropped regions or individual patches. In practical pipelines such as DeepNAPSI, models based on BEiT are adopted and have shown strong robustness under uncontrolled imaging conditions and variable lighting. This helps to guarantee a good performance in realistic scenarios where image quality or lighting is imperfect. Consequently, ViTs have started to emerge as powerful tools for recognizing subtle or diffused changes in the nail that might be missed by local convolutional neural networks. Their global perspective complements traditional convolutional approaches by capturing patterns extending beyond single nails and across the whole hand [122,123,124].

### 4.4. Segmentation and Detection Models (U-Net, Mask R-CNN, YOLO)

Segmentation and detection models form the backbone of nail analysis, as accurate location and demarcation of the nail plate, lesions, and capillary structures within the nail directly influence the diagnostic performance downstream. In practice, several variants of YOLO have been in wide use for detecting nail regions, melanonychia stripes, and multiple nails from a single image of the hand. Regarding segmentation, U-Net still remains the most common, if not the preferred, architecture for delineating psoriatic lesions, melanonychia bands, and nailfold capillaries due to its high-precision boundary rendering of clinically relevant regions. Furthermore, Mask R-CNN has found applications in more advanced NAPSI systems to enable multi-quadrant scoring and the structured assessment of the extent of nail involvement. Collectively, these models address unique nail-related imaging challenges, such as glare and curvature, overlapping nails, small sizes of lesions, and cluttered backgrounds, to ensure that features are robustly extracted and the input is reliable for diagnostic pipelines [122,123,124].

### 4.5. Transfer Learning Strategies

Recently, transfer learning has become one of the effective means to enhance AI models for nail disease detection when their datasets are either very small or imbalanced, which is a common problem in medical imaging. As large labeled nail image datasets are rare, transferring the model by reusing models already trained on big general datasets like ImageNet and then fine-tuning those on nail images helps, see Figure 5C. Well-known models include ResNet, Inception, MobileNet, and Vision Transformers, which can transfer their knowledge regarding basic features, such as edges, textures, and colors, to the nail domain. This greatly decreases the training time and increases the accuracy. Transfer learning has already shown strong performance for detection conditions such as nail melanoma from dermoscopic images and anemia from smartphone photos, even for only hundreds of training samples [125,126,127].

Transfer learning utilizes outside knowledge to circumvent the data limitations of different machine learning algorithms. Some advanced systems use these methodological strategies simultaneously, such as using well-tuned transfer learning algorithms and other transfer learning methodologies in unison to optimally maximize appropriateness for real-world applications. Additionally, transfer learning methodologies can be very useful for interventions in mobile health through immediate computation limitations, wherein training from the ground up is not feasible. Consequently, transfer learning approaches are critical in mobilizing AI models for various nail-based diagnostics. These AI models are effective primarily through the use of increased accuracy, generalization between devices and populations, and impunity in data-poor settings, moving nail imaging research towards scalable, effective clinical care [128,129]. A comparative overview of these models, their mechanisms, advantages, and diagnostic use cases is provided in Table 3.

## 5. Disease-Specific AI Applications Using Nail Images

Building on the imaging and AI/ML foundations outlined earlier, the actual impact of nail-based diagnostics emerges when applied to specific diseases. Nails reflect systemic, dermatological, and metabolic conditions through distinct visual and structural changes. This section illustrates case studies in which AI/ML have been applied to discover, define and monitor the various diseases from the imaging features shown by the nails. Each example will emphasis a disease, whether diabetes, anaemia, psoriasis, melanoma or onychomycosis and will outline the imaging techniques, the AI models used, the clinical relevance and the findings of contemporary studies.

### 5.1. Diabetes Analysis Based on Nail Images

The structure and chemistry of nails have the potential to reveal important changes in the body over time, providing a useful and noninvasive tool for the diagnosis of chronic diseases such as diabetes. Since diabetes is affecting the blood vessels, metabolism, and tissues of the body, it may be reflected in changes to the shape and color of nails, capillary patterns, and chemical content. Using AI and machine learning, these subtle features of nails, including color, texture, and capillary structure, are now able to be automatically analyzed using techniques such as nailfold capillaroscopy, standard photography, or laser-based imaging. Coupled with image processing methods such as segmentation or spectral analysis, these techniques have great potential in diagnosing diabetes early, monitoring the severity, and complications including foot ulcers, vascular complications, or gestational diabetes [130].

**Figure 6:**
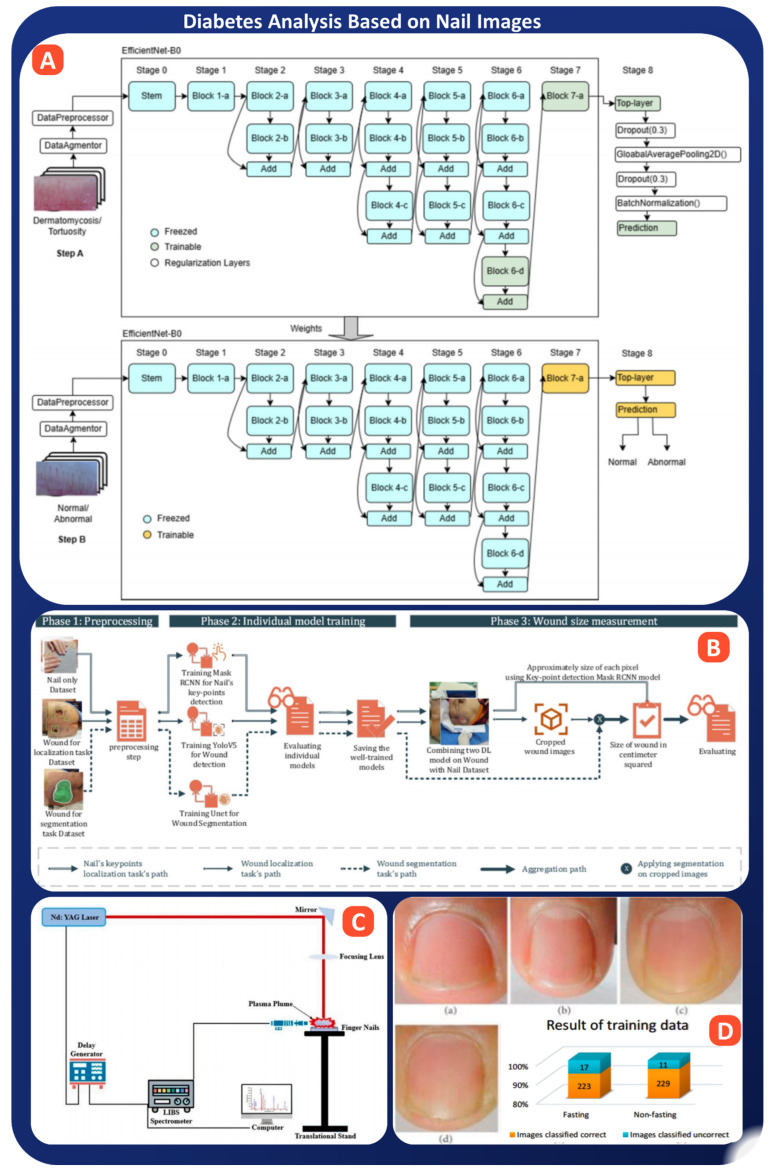
Multi-modal framework for diabetes analysis based on nail images, integrating deep learning, spectroscopy, and clinical visual evaluation. (**A**) EfficientNet-based architecture for classifying tortuous vs. normal nail patterns using dermatoscopic features, taken from [130] with the permission of Wiley. (**B**) Deep learning pipeline for wound segmentation and measurement via key-point detection and UNet fusion, taken from [132], with the permission of Springer. (**C**) LIBS (Laser-Induced Breakdown Spectroscopy) setup using Nd:YAG laser for elemental nail profiling, taken from [133] with the permission of Elsevier. (**D**) Representative nail images and training data distribution, highlighting classification performance under fasting and non-fasting conditions, taken from [135] with the permission of Elsevier.

Multiple recent investigations have illustrated the feasibility of utilizing AI-assisted nail analysis to facilitate diabetes diagnosis, employing different imaging modalities and ML models. Shah et al., performed a proof-of-concept study using CNNs on over 5000 nailfold capillary (NFC) images from 120 patients. Their models predicted diabetes status with an AUROC of 0.84, and even identified the presence of cardiovascular (CV) complications in diabetics with modest accuracy (AUROC 0.65), highlighting the broader application of NFC-based risk stratification [130]. Expanding on capillaroscopy-based approaches, Jalal et al. adopted a cascade transfer learning framework built on EfficientNet-B0 to classify NFC images as normal or abnormal (see Figure 6A). Their system achieved perfect classification scores (accuracy, precision, recall, F1 = 1.0), demonstrating not only the power of pre-trained architectures in medical imaging but also the feasibility of using NFC for routine screening of microvascular abnormalities linked to diabetes and related disorders [131]. While the above studies focus on microvascular diagnostics, Chang et al. proposed a creative application using fingernails as natural reference markers to estimate diabetic wound size in foot ulcers. Their integrated pipeline, using Mask R-CNN for keypoint detection, YOLOv5 for wound localization, and U-Net for segmentation, achieved >95% accuracy in wound measurement, shown in Figure 6B. This approach eliminated the need for manual calibration tools and provided a highly usable solution for home care and telemedicine scenarios [132]. Shifting from image-based methods to spectroscopic analysis, Rehan et al. introduced a LIBS (Laser-Induced Breakdown Spectroscopy)-based framework to analyze chemical signatures in fingernails of diabetic vs. non-diabetic individuals, as shown in Figure 6C. Using ensemble and stack learning across 4800 spectra, their model achieved outstanding results, accuracy of 96%, precision of 99.9%, and sensitivity of 96.7%, showing that diabetes leaves a traceable elemental fingerprint on nail keratin [133]. A related study by Carter et al. employed ICP-MS and MIP-OES for elemental profiling of toenails, followed by ML for classification. Using RF with selected trace elements (Al, Zn, Cu, etc.), their model achieved an AUC of 0.90 on test data, highlighting the practicality of mail-in nail-based diagnostics for patients in remote or underserved areas [134].

Turning to image classification, Kurniastuti et al. examined basic neural networks trained on gray scale images of fingernails along with texture features derived from GLCM. Their model achieved 100% accuracy in fasting conditions and 85% accuracy under non-fasting conditions. This is indicates strong signal retention through the use of basic image preprocessing and basic architectures (Figure 6D). This shows promise for highly accessible mobile screening tools [135]. Extending the concept to gestational diabetes, Chan et al. analyzed the fingernail elemental content of pregnant women and applied an ensemble ML model for early prediction of gestational diabetes mellitus. Their model, trained on Ni, Cu, and Se levels, achieved an AUC of 0.81 and was validated on an external dataset with an AUC of 0.71, establishing nails as valuable biomarkers even before disease onset [136].

The studies of AI-driven nail diagnostics indicate a mid-level stage of readiness, yet strong potential for real-world application using nail images to detect diabetes. Various proof-of-concept models (TRL 3–5), including CNNs, EfficientNet, U-Net, and spectroscopic-based methods such as LIBS and ICP-MS, demonstrate high accuracy (mostly over 90%) as proof that subtle structural and chemical changes in nails can reflect diabetic conditions, making them reliable non-invasive markers for the early detection and monitoring of diseases. Yet, several challenges remain to be addressed: small and non-diverse datasets, variability in lighting and pigmentation, and lack of standardized imaging protocols. Larger multicenter studies and prospective trials will be required to achieve full clinical scaling. The most practical near-term applications are smartphone-based self-screening, telemedicine tools, and community-level mail-in assays. With continued progress in explainable AI, federated learning, and mobile health systems, a new generation of nail-based diabetes diagnostics may soon become viable, cost-effective, scalable, and patient-centric solutions for healthcare.

### 5.2. Hemoglobin Analysis Based on Nail Images

Hemoglobin, the protein in red blood cells, carries oxygen and is a very important marker for anemia and its related disorders. Traditional methods of measuring hemoglobin involve drawing blood and techniques such as the cyanmethemoglobin test, which can be cumbersome, expensive, and painful, and also inaccessible in low-resource settings [137]. Clinicians have long used nail pallor as a visual cue indicative of anemia, since this reflects oxygenation status and altered microvasculature. Today, through AI and machine learning, researchers are developing non-invasive means of assessing anemia from photos or videos of the nailbed, occasionally under gentle pressure, to estimate hemoglobin levels. Such models utilize features like nail color, the filling rate after pressure is applied, and light reflection patterns to enable fast, portable, and user-friendly diagnostics with the use of smartphones or small devices. The nailbed is particularly fitting because it has very little melanin and an excellent view of blood vessels [138].

**Figure 7:**
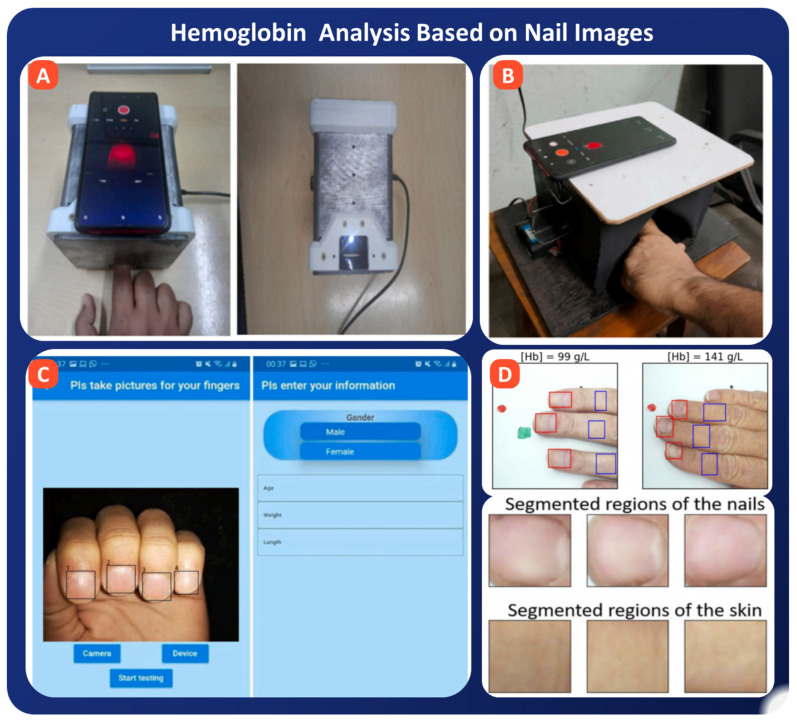
Hemoglobin analysis based on nail and skin images using smartphone-assisted imaging and AI-driven segmentation. (**A**) Prototype 1: Smartphone-based nail imaging setup with finger positioning for hemoglobin estimation, taken from [139] with the permission of Elsevier. (**B**) Prototype 2: Enclosed box design for ambient-light-free nail image acquisition using a smartphone, taken from [140], with the permission of Elsevier. (**C**) Mobile application interface for user input and guided nail image capture with gender and biometric data entry, taken from [141] with the permission of Elsevier. (**D**) Sample outputs showing segmented nail and skin regions from subjects with low (99 g/L) and normal (141 g/L) hemoglobin levels, indicating region-wise analysis for hemoglobin prediction, taken from [142] with the permission of Springer.

Many AI-powered approaches have demonstrated promising results in non-invasive hemoglobin estimation using nail images or videos. Das et al. proposed one of the most advanced systems, which developed an embedded, automated nail device coupled with a GNN-based deep learning pipeline (AneumoNet + multi-head attention regression), shown in Figure 7A. Their system achieved 94% prediction accuracy and 97% classification accuracy on 644 samples, with RMSE = 0.43 g/dL, marking a new benchmark in non-invasive Hemoglobin diagnostics [139]. In a separate yet complementary study, the same group introduced the ADKit_Nail framework, which used a fusion of ensemble regression models and a multilayer perceptron to process video data of nail pallor changes under pressure, as shown in Figure 7B. Their system, tested on 220 subjects, achieved RMSE = 0.63 and a standard deviation of ±0.46 g/dL, illustrating robust performance even in resource-limited conditions [140]. Meanwhile, Yılmaz et al. proposed a combined deep learning model that processed nail images and demographic variables (age, BMI, gender) from 353 participants, as shown in Figure 7C. Their approach yielded RMSE = 0.56 g/dL, bias of 0.03 g/dL, and MAPE = 2.09%, offering a highly accurate and ultra-fast alternative with an average processing time of just 0.09 s [141]. Yakimov et al. made a critical infrastructural contribution by publishing an open dataset of 250 patients’ RGB nail and skin images paired with certified Hb values (See Figure 7D). This dataset, balanced for age, gender, and skin tone, is a benchmark for the community to validate and standardize models under real-world diversity [142].

In light of recent research and comprehensive evaluations of AI-enabled nail diagnostics, hemoglobin estimation from nail images shows strong translational promise at a mid-level of technical readiness. Advanced pipelines using CNNs, YOLO-based detection, and ensemble regression have achieved impressive performance, with accuracies above 90% and RMSE values as low as 0.4–0.6 g/dL. Smartphone-based imaging systems, coupled with preprocessing and segmentation, further enable rapid, user-friendly assessment of hemoglobin, aligning well with point-of-care and telemedicine needs. Notably, the creation of open datasets that connect nail images with certified hemoglobin values has enhanced the benchmarks and validations possible in this field. However, additional challenges must be addressed before this technology can be applied in real-world situations. Differences in lighting, camera quality, and diversity of populations can affect the fine color features and the reliability of model performance. Solutions to these problems will include standardized imaging methodologies, larger and more diverse datasets, and the application of explainable AI technologies. With these solutions, nail image-based hemoglobin testing may become a practical, non-invasive, and useful means of diagnostic testing, particularly in children and in low-resource settings.

### 5.3. Psoriasis Analysis Based on Nail Images

Nail psoriasis is a very common and obvious manifestation of psoriasis, occurring in almost half of the patients suffering from skin psoriasis and up to 90% of those with psoriatic arthritis. It causes pitting, crumbling, yellow-brown “oil-drop” spots, thickening, and other changes in the nail plate or nail bed. These impair not only appearance but also fine motor skills and quality of life. Its severity is usually assessed by doctors using systems such as NAPSI or mNAPSI, but this is time-consuming and can be variable between observers. Several groups have therefore developed AI-based image analysis tools that automatically detect nails, identify disease features, and estimate NAPSI scores close to human expert performance. Such tools might prove clinically useful, offer follow-up benefits for treatment evaluation, and even allow for self-examination by the patient via a smartphone app [143,144].

**Figure 8:**
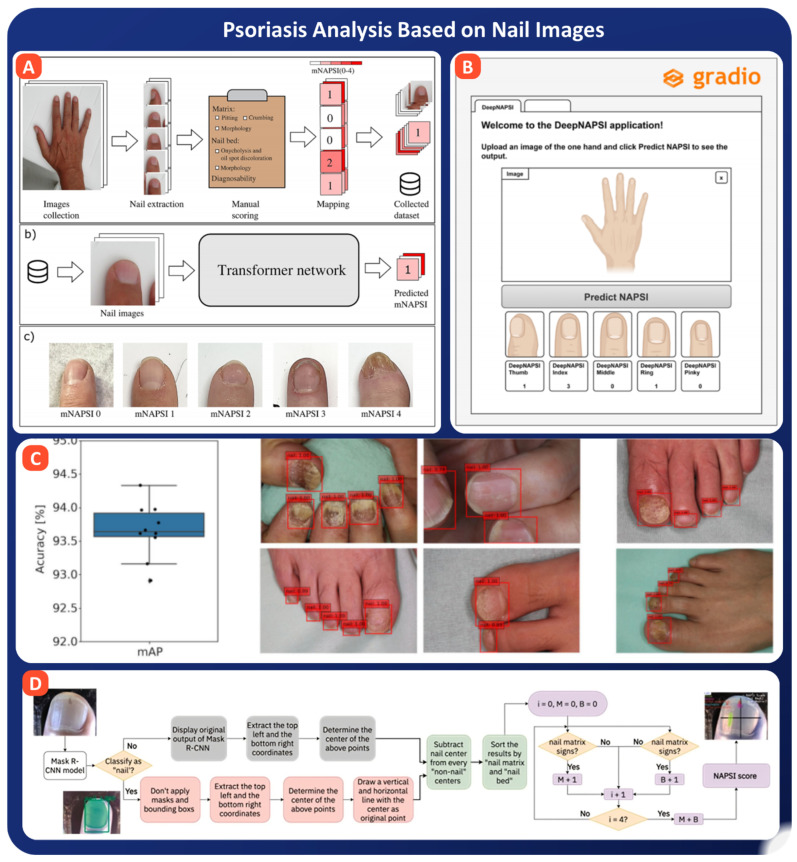
Automated psoriasis detection and scoring using nail image analysis. (**A**) Workflow for dataset creation and prediction: includes nail image collection, manual mNAPSI scoring, dataset mapping, and transformer-based prediction model, taken from [145] with the permission of Frontiers. (**B**) Gradio-based DeepNAPSI application interface for predicting mNAPSI scores from uploaded hand images, taken from [146]. Copyright Springer. (**C**) Evaluation of model performance using mAP and sample visualizations showing automated detection and scoring of nail psoriasis from real-world hand and foot images, taken from [147], Copyright Springer. (**D**) Flowchart depicting stepwise algorithm for nail region identification using Mask R-CNN, bounding box extraction, center detection, nail matrix/bed separation, and automated NAPSI score computation, taken from [148], with the permission of Elsevier.

One of the most rigorously validated systems is from Kemenes et al., who developed a CNN based on the BEiT (Bidirectional Encoder representation from Image Transformers) architecture, trained on 4400 nail photographs from psoriasis, PsA, and healthy individuals (see Figure 8A). Their model achieved a mean AUROC of 86% on training data and 80% on an independent validation set, with a strong patient-level Pearson correlation of 0.94, demonstrating both accuracy and robustness across variable imaging conditions [145]. Complementing this, Folle et al. proposed DeepNAPSI, another BEiT-based model that used hand photographs and automatic key-point extraction to locate individual nails, as shown in Figure 8B. Their tool achieved an AUROC of 88% and PR-AUC of 63%, with open access to the entire system, promoting clinical reproducibility and integration [146]. To reduce variability and enhance clinical adoption, Horikawa et al. designed the “NAPSI Calculator” using a deep learning pipeline that accurately scored 137 out of 138 nails with 83.9% accuracy, statistically outperforming both dermatology residents and board-certified physicians, as shown in Figure 8C [147]. Further advancing automation, Hsieh et al. built a standardized imaging system integrated with Mask R-CNN for segmentation and scoring, as shown in Figure 8D. Their system predicted individual nail NAPSI scores and reduced doctor workload by enabling automated assessments from routine images [148].

Meanwhile, Jia et al. introduced a cascaded deep learning pipeline comprising nail detection, lesion segmentation, and quadrant classification models for Target NAPSI scoring. Though still in early stages, their system achieved quadrant classification accuracy of 76.5% and is under ongoing refinement [149]. Beyond AI-assisted scoring, risk stratification of nail involvement is emerging as an essential step for preventive dermatology. Peng et al. developed a nomogram based on a nationwide cohort of 3920 patients in China. Using demographic and clinical features, they created a logistic regression model that predicted nail involvement risk with an AUROC of 0.745. This tool helps prioritize high-risk patients for nail-specific screening and early intervention [150].

Following studies on diabetes and hemoglobin, the analysis of psoriasis through images of nails demonstrates the versatility of AI-assisted diagnostics. Transformer-based and CNN models trained on thousands of nail photographs exhibit AUROC values of 80–88% which closely match those recorded by the dermatologist for the corresponding NAPSI score. Such systems as DeepNAPSI and “NAPSI Calculator” illustrate how automated pipelines decrease inter-observer variability and provide a uniform severity rating. Mobile and web-based tools extend this capability to teledermatology and self-assessment. The results are promising, but correspondingly extensive validation is needed in heterogeneous populations in a wide range of imaging conditions and clinical workflows before implementation in routine care.

### 5.4. Anemia Analysis Based on Nail Images

Anemia, especially iron-deficiency anemia (IDA), remains one of the most significant health problems worldwide, affecting many children, women of reproductive age, and underserved populations. It lowers hemoglobin levels, reducing the blood’s capacity to carry oxygen, which can provoke fatigue, poor concentration, and developmental delays. Classically, the diagnosis of anemia required blood tests and laboratory facilities, often inaccessible in low-resource settings and for children. Hence, driven by advances in AI and computer vision, there is a growing interest in researching new non-invasive methods to detect anemia by analyzing pallor of the nails, a long-recognized anemia symptom. Modern machine learning models such as deep CNNs and ensemble methods study the color, texture, and light reflection from nails to estimate hemoglobin levels without blood draws. Imaging-based methods are especially promising because they are accessible through smartphones, painless, and applicable for real-time screening in schools, homes, and remote clinics [151,152].

**Figure 9:**
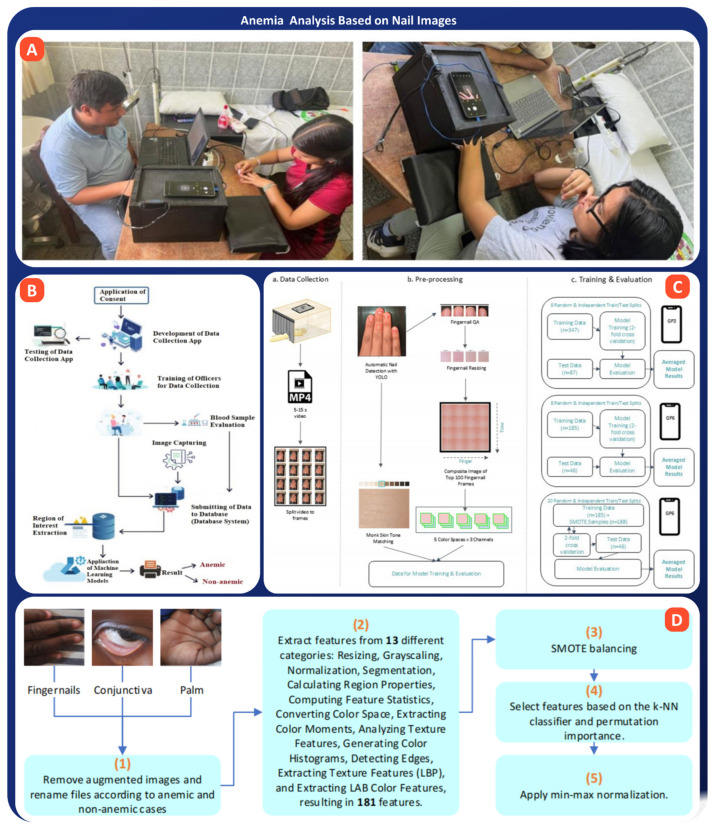
Workflow for anemia detection using nail image analysis. (**A**) On-site data acquisition setup showing smartphone-based nail image capture integrated with computer-based data entry, taken from [39], Copyrights Frontiers. (**B**) Data collection and processing pipeline, including app development, officer training, consent application, image capturing, blood sampling, and database integration, taken from [44], Copyrights Wiley. (**C**) Model training and evaluation framework involving YOLO-based nail detection, pre-processing (e.g., color space transformation and resizing), and multi-split evaluation with CNN models, taken from [45], Copyrights Sage. (**D**) Feature engineering steps, including removal of augmented images, extraction of 181 features from 13 categories (e.g., color, texture, edge, LAB space), SMOTE-based class balancing, feature selection using k-NN with permutation importance, and min-max normalization for training robust classification models, taken from [153], Copyrights MDPI.

Several recent studies have demonstrated significant potential for the detection of nail image-based anemia. Navarro-Cabrera et al. conducted a study involving 909 university students and trained three deep learning models, DenseNet169, InceptionV3, and Xception, on smartphone-captured nail images, as shown in Figure 9A. Among them, DenseNet169 performed best, achieving an accuracy of 69.83% and an AUC of 0.74. The model correlated well with Rad-67 hemoglobin meter readings, underscoring the viability of image-based screening in young adult populations [39]. In another comparative study, Asare et al. evaluated five machine learning algorithms (Naïve Bayes, CNN, SVM, k-NN, and decision tree) using images of fingernails, palms, and conjunctiva from 10 hospitals in Ghana, as shown in Figure 9B. CNN outperformed all models with 99.12% accuracy, while fingernail images emerged as the most predictive site for anemia detection [44]. The HEMO-AI study by Gordon et al. developed a smartphone-based screening tool tailored to pediatric patients. Their ML model, trained on 823 annotated samples from children aged 6 months to 18 years, achieved 87% sensitivity and 84% specificity using fingernail image features extracted with YOLOv8 and XGBoost, laying the groundwork for a non-invasive, pediatric-specific anemia tool (See Figure 9C) [45]. Berghout et al. furthered this work with a deep recurrent expansion network (RexNet) used on pediatric fingernail, palmar, and conjunctival images. RexNet was able to achieve 99.83% on all metrics and showed greater interpretability of the models, indicating its readiness for clinical application (see Figure 9D) [153]. Further attempting to quantify anemia types, Saputra et al. have proposed using an Extreme Learning Machine to categorise Iron Deficiency Anemia, Beta Thalassemia Trait and Hemoglobin E from information given from images and hemotological data. They correctly classified the condition in 99.21%, suggesting that automatic feature classification is attainable [154].

Case studies demonstrate that anemia detection using nail images is already at a middle to high level of readiness, with some models already very close to being used clinically. Deep learning models like CNNs, DenseNet, and RexNet have reached very high accuracy, between 95–99%, while pediatric tools such as HEMO-AI showed sensitivity and specificity of more than 85%, proving they could work well in real-world settings. With smartphone-based image capture and preprocessing, hemoglobin levels can be estimated in minutes, even enabling school or community screenings in low-resource settings. The release of open datasets linking nail images to certified hemoglobin values has further improved the validation of such methods. However, challenges regarding the stability of different light conditions, the modification of nails according to age, and limited population diversity remain. Such issues need to be resolved before these non-invasive tools can see wider application.

### 5.5. Melanoma Analysis Based on Nail Images

Melanoma is the most dangerous form of skin cancer, and its nail variety, named nail apparatus melanoma, is particularly serious due to its late diagnosis and rapid dissemination. The common initial symptom is a dark streak on the nail, called melanonychia; however, this may also be caused by harmless conditions, such as moles, fungal infections, or bleeding [155]. Although biopsy is still the most reliable confirmation method, it is an invasive, painful procedure that can result in lasting damage. On account of the rarity of nail melanomas and their often-unusual appearance, especially in individuals with darker skin, there is an overwhelming need for safe and non-invasive screening. Advances in AI and deep learning now enable image analysis of nails captured by either dermoscopes or smartphones. Systems based on image segmentation, lesion tracking, and risk scoring-including clinical rules like ABCDEF-offer accurate, explainable, and accessible tools for melanoma detection and monitoring [156,157].

**Figure 10:**
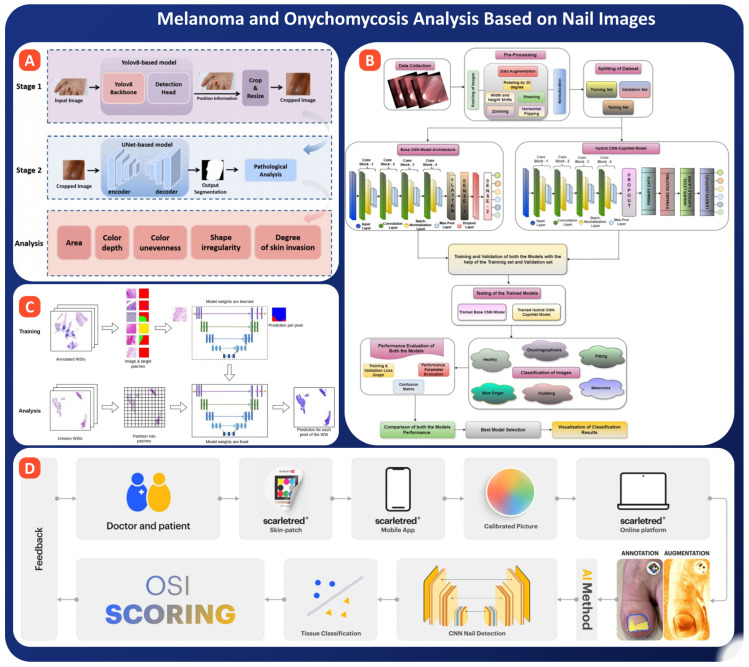
Deep learning-based frameworks for melanoma and onychomycosis analysis using nail images. (**A**) Two-stage pipeline: YOLOv5-based object detection localizes and crops nail regions, followed by U-Net-based segmentation for pathological analysis, taken from [158] with the permission of Elsevier. (**B**) Comparative training pipeline involving base CNN and hybrid CNN-detection and U-Net segmentation have achieved high F1 and Dice scores, enabling accurate lesion localization and ABCDEF rule-based risk stratification. Interpretability-focused systems further align outputs with dermatologists’ assessments, supporting trust in clinical use. However, sensitivity remains lower for subungual melanoma compared to other skin cancers, highlighting the need for subtype-specific training and larger datasets. Practically, AI-powered mobile or dermoscopic tools could support early triage, but regulatory validation and prospective trials remain essential capacitated models, with pre-processing techniques (e.g., zooming, resizing, denoising), training-validation split, and performance evaluation across multiple nail diseases (onychomycosis, clubbing, pitting, melanoma), taken from [161], with the permission of Springer. (**C**) U-Net-based histopathological segmentation framework: trained on annotated patches and tested on whole-slide images to predict disease-specific pixel-wise features, taken from [162] with the permission of MDPI. (**D**) scarletred^®^ AI-powered platform workflow: integrates mobile imaging, calibrated scoring, CNN-based nail detection, tissue classification, and augmented feedback to physicians for enhanced diagnostic support, taken from [163], Copyrights Wiley.

Recent studies demonstrate that deep learning models can help in the early detection of melanoma by using nail images. Weng et al. proposed a two-step system for melanonychia analysis: nail detection with YOLOv8 and lesion segmentation with UNet. The system was trained using 200 smartphone images taken at Sun Yat-sen University hospitals and demonstrated outstanding results: the DBB variant of YOLOv8 obtained an F1 score of 0.986, the Dice score for the UNet model with receptive field attention was 0.728, and specificity was 0.939 (see Figure 10A). An ABCDEF rule-based risk tool has classified the detected lesions into four stages of severity, with close correspondence to pathological examination. There is a great prospect of putting this approach into real diagnostic practice [158].

In this complementary study, Chen et al. built a model for performing interpretability analysis of pigmented nail lesions using U-Net segmentation and features of images inspired by ABCDEF: area ratio, mean pixel intensity, evenness, irregularity and invasion into surrounding skin. These indicators are found to correlate well with the assessments made by dermatologists (e.g., R^2^ = 0.82 area ratio as compared to breadth score). This shows that the model segments the images well, but also gives interpretable outputs of outputs of risk [159]. Winkler et al. applied a commercial CNN for our benchmarking (Moleanalyzer-Pro^®^) to six subtypes of melanoma, including 30 subungual melanoma cases. The performance of the CNN was excellent for other localizations (AUC > 0.92), but was particularly disappointing for nail melanoma with a sensitivity of 53.3% and an AUC of just 0.621. This suggests the need for subtype-specific training [160]. Finally, Melanoma detection through nail images is at an early-to-mid technical readiness level, with case studies showing both promise and challenges. AI models combining YOLO-based.

### 5.6. Onychomycosis Analysis Based on Nail Images

Onychomycosis is a common and chronic fungal infection of the nails, making up roughly half of all nail diseases. It often causes nail discoloration, thickening, and damage that can affect appearance and quality of life because of social stigma. Conventionally, it is diagnosed by clinical examination, KOH microscopy, fungal cultures, or tissue stains; however, these conventional techniques can be time-consuming, less accurate, and highly dependent on the examiner’s skills. With recent advances in digital imaging and AI, new non-invasive methods have emerged. By analyzing nail texture, color, and shape from smartphone photos or images taken in the laboratory, deep learning models are now able to detect fungal infections more quickly and even measure disease severity and treatment progress [24].

Recent studies have highlighted AI’s growing role in automating onychomycosis diagnosis. Shandilya et al. developed a hybrid deep learning architecture combining CNN and Capsule Networks (CapsNet) to classify six nail conditions, including onychomycosis, using 3835 images from the Nail Disease Detection dataset, as shown in Figure 10B. The hybrid model achieved 99.25% validation accuracy, with a precision of 97.35% and recall of 96.79%, outperforming the baseline CNN model and demonstrating improved spatial representation using dynamic routing [161]. Jansen et al. took a histopathology-based approach by developing a U-NET model trained on 664 PAS-stained whole-slide images (WSIs) from four different labs. Their model achieved a 90.5% detection rate for fungal elements, matching the sensitivity of 11 experienced dermatopathologists (See Figure 10C). With real-world diversity in staining protocols and slide digitization, their model demonstrated clinical robustness and potential as a diagnostic aid [162]. In a clinical image study using CE-certified, self-calibrating technology, Agostini et al. took 1687 images from 440 subjects using the Scarletred^®^Vision app. Their AI model was trained on the images and obtained an F1 score of 0.8566 for toenail segmentation and a classification accuracy of 81% with a RF model, as well as dynamic tracking of disease progression employing colour and texture segmentations (see Figure 10D). The authors described one of the systems created to quantify the severity of onychomycosis by the use of AI over time [163].

Analysis of onychomycosis, fungal infection of the nails, using nail images is at a mid-to-high level of readiness; most studies have demonstrated that it could work well in actual clinical settings. Such tools could support doctors in making decisions, teledermatology screening, and follow-up of treatments over time. However, for their more extensive application in clinical practice, this will require standardization of imaging methods, larger and more diverse datasets, and more comprehensive validation, specifically to manage nail thickness variations, light, and staining, which vary from one clinic to another. With more development and approval by regulatory bodies, AI-based analysis of onychomycosis might prove to be an efficient, non-invasive, and scalable tool for both hospitals and community health programs. The following comparative table shows overview of disease-specific applications, technical readiness levels, key case study outcomes, and real-world implications is presented in Table 4 which consolidates findings across diabetes, hemoglobin estimation, psoriasis, anemia, melanoma, and onychomycosis [164].

Finally, Section 5 demonstrates that AI-powered nail diagnostics are increasingly moving from theoretical demonstrations to tools that can be used in real clinical settings, with clear, category-specific progress across different disease areas. Regarding the studies on the estimation of diabetes and hemoglobin, the main point deduced was that subtle color changes and vascular patterns within the nail—patterns previously too faint to be readable manually—could now be quantified, using advanced machine learning approaches including CNNs, EfficientNet architectures, and ensemble regression methods. These approaches reach accuracies routinely well over 90%. This body of evidence underlines that the nail bed is a physiologically rich substrate capable of reflecting the systemic metabolic state with notable precision, especially when combined with extensive preprocessing and accurate region-of-interest segmentation.

Transformer-based models, such as BEiT and DeepNAPSI, combined with hybrid CNN-CapsNet architectures, for skin disorders like psoriasis and onychomycosis, lead to a significant reduction in inter-observer variability and support more objective disease scoring. Evidence exists that AI is capable of much more beyond emulating expert-level assessments: it enforces standardized quantitative scoring work processes. In this process, it helps to reduce one important potential clinical dermatology limitation by providing consistent and reproducible assessments. In the domain of melanoma analysis, good results regarding segmentation and lesion detection using YOLO have been observed; this is evident from high F1 and Dice scores. However, the most critical conclusion is that AI models still struggle to detect subungual melanomas with a high degree of certainty. This finding suggests the requirement for subtype-specific training and building larger, more thoroughly curated datasets. Similarly, few studies of anemia classification report very high accuracy of up to 99%. Yet, this performance is not consistent; it varies based on factors like lighting conditions and skin pigmentation. This variability underlines how important it is to ensure the diversity of the dataset and applies the illumination-invariant preprocessing methods.

Taken together, these results suggest that AI models performed well in diseases with consistent, easily recognizable structural or chromatic cues-for example, anemia, psoriasis, and onychomycosis-and underperform in conditions where the visual signals of the disease are usually subtle, highly variable between cases, or not well represented in the training data, as is common with melanoma. The evidence across studies supports a clear path toward improvement: multi-center datasets should be larger; imaging protocols should be standardized; and models must output clinically interpretable results. Proceeding with these steps will be essential to transform AI-enhanced nail diagnostics from mere research prototypes into scalable, equitable point-of-care tools.

In last, we have prepared an integrated overview to present a complete, unified view of the resources already available for AI-assisted nail diagnostics research. To put all the essential details in one place, Table 5 summarizes the main characteristics of the datasets that have been most widely used in recent studies, including size, imaging modality, and the spectrum of represented pathologies. These datasets represent a wide range of imaging modalities, from smartphone-based RGB photography to dermoscopy, further to nailfold capillaroscopy and histopathology. Although there is an increasing interest in the area, most of the datasets are still relatively small in size; they are collected within one institution, and only a limited number of these are publicly available-for instance, the works of Yakimov et al., among few others. This summarizes both the progress that has been achieved and the important gaps that persist. In particular, it underscores ongoing issues related to dataset diversity, standardization, and scale. These are central challenges in that they have direct bearing on the generalization of AI models, translation of said tools into clinical practice in a timely manner, and vice versa. The integrated summary of available datasets for AI-Assisted nail diagnostics is provided in the following Table 5.

## 6. Key Challenges and Possible Solutions

Although significant progress has been made in AI-assisted nail diagnostics, there are significant challenges to adoption in clinical practice and scalability in the real world. These include technical challenges such as a lack of data, variability in image acquisition results, but also global challenges such as model interpretability, clinical validation or generalisability across different populations and devices. The next subsections seek to systematically answer these challenges, revealing potential solutions to improve the robustness, fairness and translational potential of AI-based nail disease detection systems.

### 6.1. Limited, Diverse Datasets Across Populations

Most nail datasets are small, single-center, and lack diversity in skin tone, nail morphology, disease stages, and imaging devices. This results in overfitting, biased predictions, and poor generalization outside controlled environments [167,168].

**Targeted Solutions:**

(1) Develop multi-institutional datasets modelled after ISIC, with standardized imaging protocols and metadata (device, lighting, Fitzpatrick scale) [169,170].
(2) Use federated learning so models can learn from distributed clinical data without moving patient images.
(3) Employ GANs and domain-randomization only as augmentation tools, not substitutes, especially to simulate variations in tone, curvature, glare, and pathology patterns [171,172].

### 6.2. Variability in Lighting, Nail Color, Skin Tone

RGB nail images vary significantly due to lighting, camera quality, reflections, and skin pigmentation. These variations disproportionately affect tasks dependent on fine color cues, such as anemia or melanoma detection [15,17,173].

**Targeted Solutions:**

(1) Integrate device-agnostic preprocessing pipelines (white balancing, illumination correction, specular-reflection removal).
(2) Use color-calibration markers or controlled illumination modules in smartphone-based capture systems.
(3) Train models with stratified datasets across skin tones to ensure fairness and reduce bias [174,175,176].

### 6.3. Annotation Errors and Absence of Clinical Labels

Nail images often lack dermatologist-verified labels. Mislabeling (e.g., confusing benign pigmentation with melanoma or fungal infection) propagates systematic error into the model [177,178].

**Targeted Solutions:**

(1) Adopt multi-reviewer annotation workflows and consensus scoring for segmentation and disease labels.
(2) Use weak-supervision and label-noise-robust training to handle imperfect labels.
(3) Encourage structured clinical reporting at data collection (NAPSI score, disease subtype, severity grade) [179,180,181].

### 6.4. Model Interpretability and Clinical Validation

As AI/ML is incorporated into clinical workflows, model explainability and thorough clinical validation become paramount, particularly in sensitive areas such as nail disease detection. Notwithstanding excellent benchmark performance, most deep learning models remain “black boxes,” thus limiting trust, uptake, and regulatory approval of the models. In nail diagnostics, where clues such as pigmentation, ridging, or pallor may be subtle, clinicians require information on the regions or features of the images which influenced the AI decisions. Without visual explanations, the AI predictions may be inconsistent with the clinical diagnosis, generating skepticism, particularly with multi-class or grades of severity predictions [182,183].

**Targeted Solutions:**

(1) Use attention maps, Grad-CAM, and ABCDEF-linked feature visualization to show clinically relevant cues [184,185].
(2) Integrate rule-based modules (e.g., lesion width, color asymmetry, microvascular patterns) for hybrid interpretable outputs [186,187].
(3) Test models with physicians in controlled clinical workflows to improve usability and trust [188].

### 6.5. Generalizability of Models Across Devices and Environments

A major challenge in applying AI/ML (artificial intelligence/machine learning) analyses of nails in clinical practice is how well the models can perform on different devices, lightness, and user situations. While most perform so well on specially prepared research datasets, they often perform at a significantly reduced output when images are obtained from different smartphones, under different lighting conditions, and with different backgrounds, and in different clinical applications. This inapplicability of the training to real-world applications further reduces the reliability and scalability of analyses of nails based on AI models. The real seriousness of the challenges faced come about from the fact that the devices and environments are different, since nails can be imaged with anything from advanced dermatoscopes and OCT machines to ordinary smartphone cameras [189]. They all have pronounced variations in resolution, lens quality, sensor calibration, and color reproduction. In addition, user-controlled variables such as angle, distance, flash/intensity, and the positioning of their hands can also contribute to variation. Without standardized imaging protocols, the same nail condition can appear significantly different across devices or environments, confusing AI models and reducing prediction confidence [190].

**Targeted Solutions:**

(1) Employ self-supervised and contrastive learning to build device-robust feature representations capable of generalizing across diverse imaging sources.
(2) Use environment-specific fine-tuning on small local datasets (e.g., clinic- or device-specific samples) to reduce the performance gap during deployment.
(3) Implement federated learning to train models collaboratively across heterogeneous devices without centralizing patient images, enhancing generalization while preserving privacy.
(4) Integrate real-time image quality control within diagnostic apps to automatically detect and correct issues related to blur, lighting, framing, and angle before inference.
(5) Apply device-agnostic preprocessing pipelines (illumination correction, color normalization, geometric alignment) to reduce variability at the input stage [191,192,193].

In summary, ensuring that AI/ML models perform reliably across different devices and environmental contexts is critical for successfully translating into real-world diagnostic platforms. Addressing this challenge requires diverse data collection, robust modeling strategies, and intelligent deployment frameworks to ensure consistency, safety, and fairness in nail-based disease detection. The following Table 6 outlines key stakeholders, the challenges they can influence, and phased actions from the near term to 2035 to accelerate safe, equitable, and scalable deployment of AI-based nail diagnostics. The 2025–2035 window is used as an illustrative 10-year planning horizon, reflecting typical timelines for translating imaging AI systems from proof-of-concept to large-scale clinical deployment in related fields such as dermoscopy and retinal screening, rather than a precise prediction of specific milestone years.

## 7. Conclusions and Future Direction

This review, for the first time, comprehensively collates the entire range of AI-driven approaches for nail diagnostics in a wide array of imaging modalities, several disease categories, and a range of different computation methods. We systematically compare imaging using a smartphone, dermoscopy, OCT, capillaroscopy, and histopathology to reveal how the diagnostic value of the nail has been underestimated when compared with many other areas of dermatology and imaging. Our review not only highlights the best-performing AI models but also openly reveals the structural barriers standing in the way of practical implementation. These obstacles include limited access to diverse and large datasets, differences across measuring devices, inconsistencies in the way annotations are made, and a lack of standardized imaging protocols.

Going beyond a simple summary of existing works, we introduce a layered research framework that emphasizes the need for domain-adapted preprocessing steps, the use of interpretable model architectures, and the adoption of federated and privacy-preserving learning strategies, along with validated clinical workflows tailored for the specific needs of nail diseases. This in-depth analysis allows the review to detail the technical readiness for each disease application-diabetes, anemia, psoriasis, melanoma, and onychomycosis-and provides a structured road map intended to advance these AI systems from preliminary proof-of-concept models toward robust, real-world diagnostic tools.

In summary, this work places nails as a very promising but at the same time highly underexplored substrate for non-invasive diagnostics. Therefore, it provides targeted recommendations aimed at accelerating the maturation, enhancing fairness, and improving the clinical reliability of AI-enabled detection of nail diseases.

## Figures and Tables

**Table 1:** Comparison of Image Acquisition Techniques for Nail Analysis.

Modality	Resolution & Depth	Clinical Use	Advantages	Limitations	AI Integration Potential
Smartphone Cameras	Moderate resolution Surface-level imaging	Screening for anemia, fungal infections, and psoriasis	Ubiquitous, low-cost, easy to use, enables self-monitoring	Variability in lighting, focus, and background clutter	High (widely used in mobile AI apps, real-time processing)
Dermoscopy	High resolution Surface and subsurface visualization	Melanoma detection, psoriasis, and onychomycosis	High diagnostic clarity reveals pigmentation and vascular patterns	Requires equipment and training, may suffer from reflection and glare	Very High (deep learning models trained on dermoscopic datasets)
OCT	Very high resolution Depth-resolved cross-sectional views	Imaging nail plate, bed, and matrix Psoriasis, tumors	Subsurface imaging, quantitative analysis, good for structural details	Expensive, bulky, limited to clinical settings, and signal attenuation in nails	Moderate to High (used in segmentation and volumetric feature extraction)

**Table 2:** Comparison of Preprocessing Strategies for Nail Image Analysis.

Preprocessing Strategy	Purpose	Techniques Used	Benefits	Limitations
Color Correction	Restore the actual color of the nail and surroundings to enable color-based diagnosis.	White balance, histogram equalization, and the Gray World algorithm.	Improves color consistency across images; enhances color-based feature extraction.	Sensitive to lighting; may not work well in highly variable conditions.
Denoising	Remove unwanted noise or artifacts caused by low light, compression, or sensors.	Gaussian/Median filtering, Non-local Means, DnCNN.	Preserves important nail features; improves clarity for texture analysis.	Over-smoothing may blur diagnostic edges; performance depends on noise type.
Normalization	Standardize image intensity or pixel distribution across datasets and devices.	Min-Max scaling, Z-score normalization, contrast normalization.	Enhances model generalizability and stability; crucial for transfer learning.	May distort visual appearance if misapplied; lacks context-awareness.
Cropping and ROI Extraction	Focus analysis on the nail region while removing the irrelevant background.	Edge detection, contour analysis, and deep learning-based ROI models.	Reduces computational cost; improves model focus and performance.	Manual ROI selection introduces bias; automated methods require fine-tuning.
Segmentation	Precisely delineate the nail plate or lesions from surrounding structures.	Thresholding, edge detection, U-Net, ResU-Net.	Improves localization of disease features; enables multi-task AI models.	Sensitive to noise in classical methods; deep models require annotated data.

**Table 3:** Summary of AI/ML Models for Nail-Based Diagnostics.

Model Type	Examples	How It Works	Strengths	Limitations	Use in Nail Diagnostics
Classical ML	SVM, k-NN, Random Forest, Logistic Regression	Use manually extracted features (color, texture) and classify using distance, trees, or probability.	Simple, interpretable, fast training on small datasets.	Less effective for complex patterns; depends on feature engineering.	Used for binary classification (e.g., healthy vs. diseased); suitable for early-stage models.
Deep Learning	CNNs, ResNet, DenseNet	Learns hierarchical visual features directly from raw images through convolutional layers.	High accuracy, automatic feature extraction, suitable for complex visual patterns.	Needs large datasets; more computational resources.	Widely used for classifying diseases like psoriasis, onychomycosis, and melanoma.
Lightweight Deep Models	MobileNet	Optimized CNNs using depthwise separable convolutions for low-computation environments.	Efficient, mobile-friendly, close performance to standard CNNs.	May sacrifice some accuracy for speed and size.	Ideal for smartphone-based diagnostics and telemedicine apps.
Advanced Deep Models	Vision Transformers (ViT)	Uses self-attention instead of convolutions to model image relationships globally.	Captures global features; good interpretability with attention maps.	Data-hungry; requires high computation and pretraining.	Emerging use for subtle, diffuse nail abnormalities and lesion detection.
Ensemble Learning	Voting, Bagging, Boosting, CNN Ensembles	Combines predictions from multiple models to improve robustness and accuracy.	Reduces overfitting; increases confidence in predictions.	Computationally heavier; harder to deploy on mobile.	Effective for handling noisy data and maximizing performance in clinical applications.
Transfer Learning	Pretrained ResNet, MobileNet, ViT fine-tuning	Adapts models trained on large datasets to small, domain-specific nail datasets.	Reduces training time; works well with small datasets.	May underperform if pretraining and target tasks are too different.	Widely used for nail classification when data is limited.

**Table 4:** Comparative Summary of AI-Assisted Nail Image Diagnostics for Major Conditions (Section 5.1, Section 5.2, Section 5.3, Section 5.4, Section 5.5 and Section 5.6).

Disease/Condition	Technical Readiness Level (TRL)	Key Case Study Findings	Performance Metrics	Practical/Real-Time Implications
Diabetes	TRL 3–5 (proof-of-concept to early translational)	CNNs & EfficientNet on nailfold capillaroscopy; LIBS/ICP-MS elemental profiling	AUROC 0.84–0.90; Accuracy up to 96%	Smartphone-based screening, mail-in nail assays; early detection & monitoring in remote care
Hemoglobin (Anemia estimation)	TRL 4–6 (lab validation to early field prototypes)	CNNs, YOLO + ensemble models; RexNet & HEMO-AI for pediatrics	Accuracy > 95%; RMSE ~0.4–0.6 g/dL	Non-invasive Hb estimation via smartphone; scalable school/community-level screening
Psoriasis	TRL 4–6 (validated pipelines, pilot apps)	CNNs & BEiT transformers; DeepNAPSI, NAPSI Calculator	AUROC 80–88%; Correlation r ≈ 0.9 with physician scores	Reduces inter-observer variability; supports teledermatology & patient self-monitoring
Anemia (clinical anemia detection)	TRL 4–6 (mid-to-high readiness)	DenseNet, CNN, HEMO-AI, RexNet; multi-site pediatric & adult studies	Accuracy up to 99%; Sensitivity/Specificity > 85%	School & pediatric screening, rapid triage in low-resource settings; equitable, non-invasive tool
Melanoma	TRL 3–5 (early-to-mid, subtype challenges)	YOLO + U-Net segmentation; ABCDEF rule integration; interpretability pipelines	F1 ≈ 0.98, Dice ≈ 0.73; Sensitivity lower for subungual melanoma (≈53%)	Early triage, risk stratification; needs subtype-specific training + regulatory trials
Onychomycosis	TRL 5–7 (toward higher readiness)	Hybrid CNN–CapsNet, U-Net on histopath slides, Scarletred^®^Vision mobile app	Accuracy 81–99%; Segmentation F1 ≈ 0.86	Support for dermatologists; teledermatology triage; treatment monitoring over time

**Table 5:** Integrated Summary of Available Datasets for AI-Assisted Nail Diagnostics.

Study/Dataset (Year)	Imaging Modality	Pathology/Task	Dataset Size	Public?	Ref.
Ebadi Jalal et al., 2025	Capillaroscopy	NFC abnormality classification	225 participants	Private	[131]
Zhao et al., 2024—ANFC	Capillaroscopy + videos	Capillary morphology/flow	68 subjects; 321 images; 219 videos	Public	[165]
Yakimov et al., 2024	RGB nail/skin imaging	Hemoglobin estimation	250 subjects	Public	[142]
Das et al., 2023	Smartphone nail videos	Hb estimation	644 samples	Private	[140]
Yilmaz et al., 2022	RGB nail images	Hb regression	353 participants	Private	[141]
Navarro--Cabrera et al., 2025	Smartphone RGB	Anemia detection	909 subjects	Not public	[39]
Folle et al., 2023—DeepNAPSI	Clinical photos	Psoriasis severity	4400 images	Private	[146]
Paik et al., 2024	Clinical photos	NAPSI scoring	(7054 Images) Clinical dataset	Private	[166]
Horikawa et al., 2024	Clinical nail photos	Nail Psoriasis Severity	138 nails	Tool only	[147]
Jansen et al., 2022	PAS-stained WSIs	Onychomycosis detection	664 WSIs	Restricted	[162]

**Table 6:** Stakeholder Roadmap: Challenges & Solutions for AI-Assisted Nail Diagnostics (2025–2035).

Stakeholder	Challenges They Influence	Short Term (2025–2027) Actions	Mid Term (2028–2031) Milestones	Long Term (2032–2035) Commitments/Outcomes
Researchers & Academics	Dataset scarcity; bias; reproducibility; fragmented benchmarks	Publish multi-ethnic datasets; preregistered protocols; open baselines; XAI studies	Prospective multi-site trials; federated learning at scale; bias/robustness audits	Regulatory-grade toolkits; living benchmarks; standardized reporting templates
Dermatologists & Clinicians	Inter-observer variability; workflow fit; trust in AI outputs	Co-design capture protocols; structured labels/NAPSI; pilot decision support	Draft clinical guidelines; CME training on AI interpretation and bias	Routine AI-assisted grading/triage integrated into care pathways
Hospitals & Health Systems	Integration barriers (EHR/LIS); procurement; governance	Sandbox deployments; EHR connectors; governance committees; KPI dashboards	Scale across service lines; ROI & safety monitoring	System-wide adoption; value-based contracts; post-market surveillance
AI Developers & Data Scientists	Generalization; drift; security; explainability	Skin-tone–aware training; domain adaptation; privacy-preserving pipelines; model cards	Continuous learning with guardrails; drift detection; secure MLOps	Regulatory lifecycle management; safety cases; certified updates
MedTech & Imaging Vendors	Non-standard capture; hardware variability	Low-cost capture kits; on-device guidance; calibration targets	Regulatory-ready devices; interoperable SDKs; embedded QC	Standardized capture ecosystems; multi-modal devices (RGB + dermoscopy + spectral)
Regulators (FDA/EMA/CE/CDSCO)	Approval uncertainty; real-world evidence; fairness requirements	Early consultations; define endpoints; guidance for XAI disclosures	Adaptive approvals with RWE; harmonized pathways; monitoring	Mutual recognition; dynamic rules for continuously learning models
Payers & Insurers	Unclear reimbursement; value demonstration	Coverage-with-evidence pilots; codes; health-economic models	Conditional reimbursement tied to outcomes	Permanent reimbursement for validated use-cases
Patients & Caregivers	Usability; trust; privacy	Informed consent flows; accessible apps; privacy controls	Co-created education; patient-reported outcomes	Patient-owned records; longitudinal self-monitoring
Data Protection Authorities	Privacy risks; cross-border flows	Template DPAs; privacy-by-design audits; anonymization toolkits	Federated/cross-silo learning frameworks with oversight	Stable cross-border agreements for safe collaboration

## Data Availability

Data is included within this manuscript.
